# Supplementary figures and images for: Genome-Wide Screening of AP2 Transcription Factors Involving in Fruit Color and Aroma Regulation of Cultivated Strawberry
Source: Genes (Basel). 2021 Apr 5;12(4):530. doi: 10.3390/genes12040530 (PMC8067195; doi:10.3390/genes12040530)

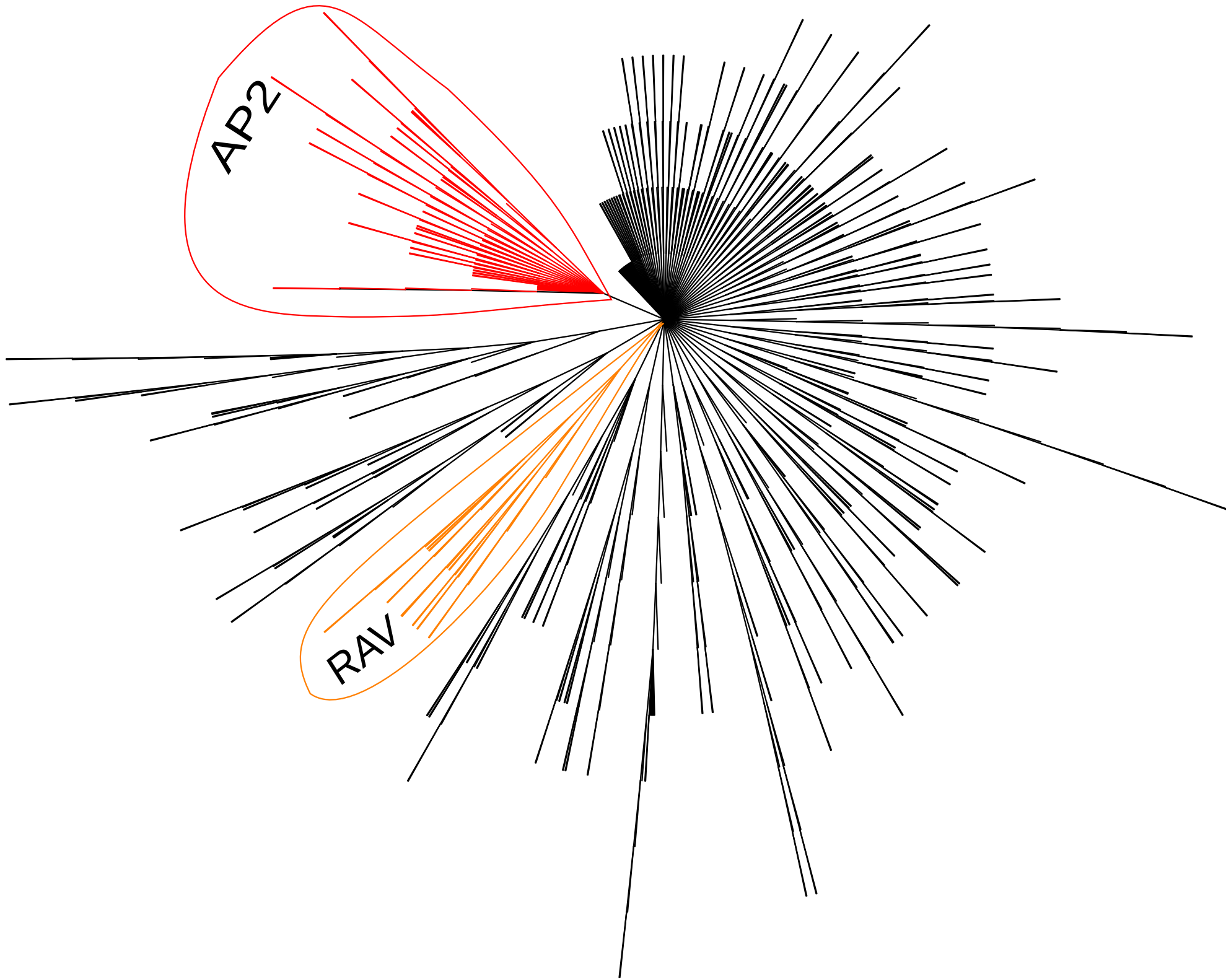

Supplement: Supplementary file 1 [file genes-12-00530-s001.zip › Figure S1.pdf]

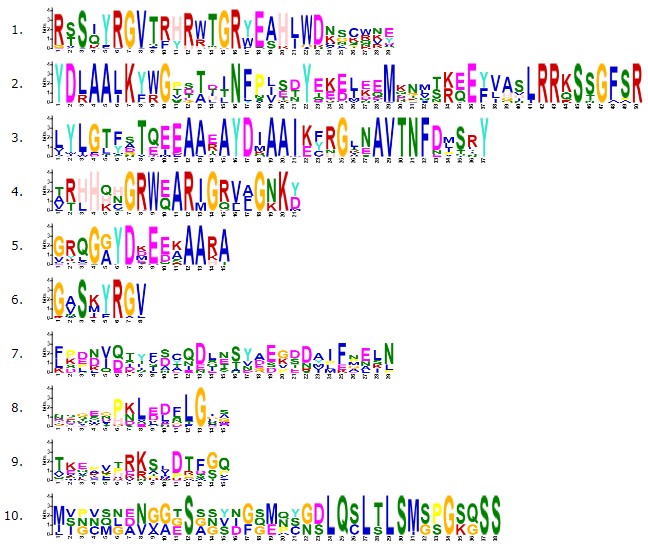

Supplement: Supplementary file 1 [file genes-12-00530-s001.zip › Figure S2.jpg]

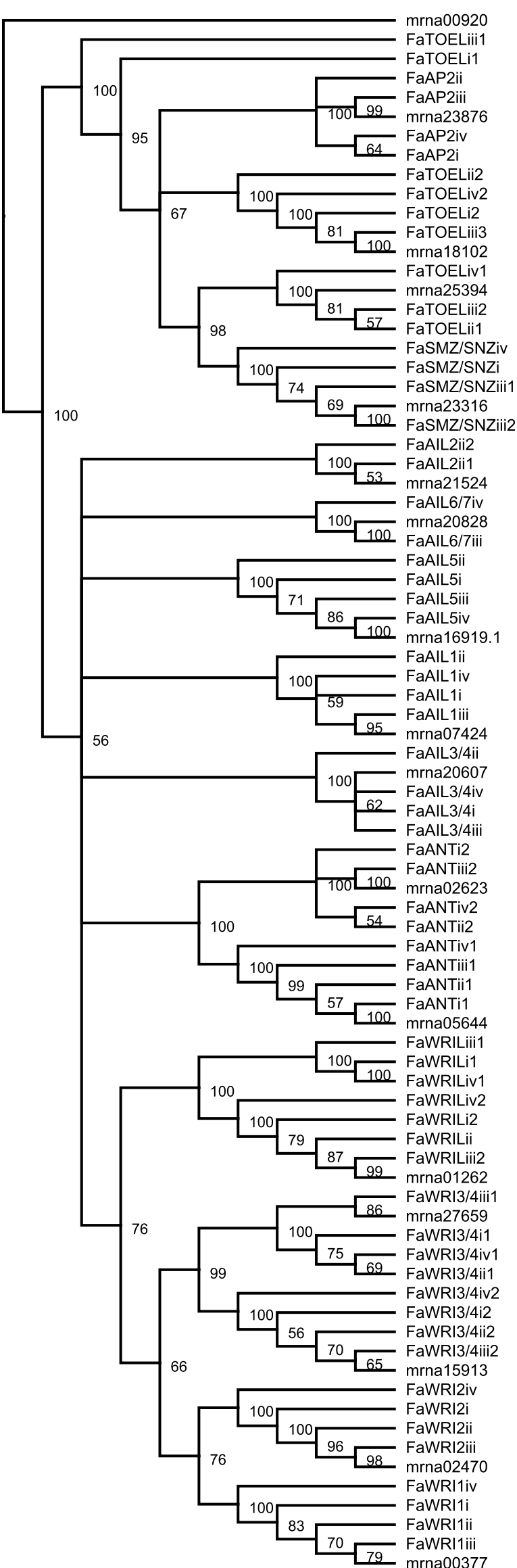

Supplement: Supplementary file 1 [file genes-12-00530-s001.zip › Figure S3.pdf]

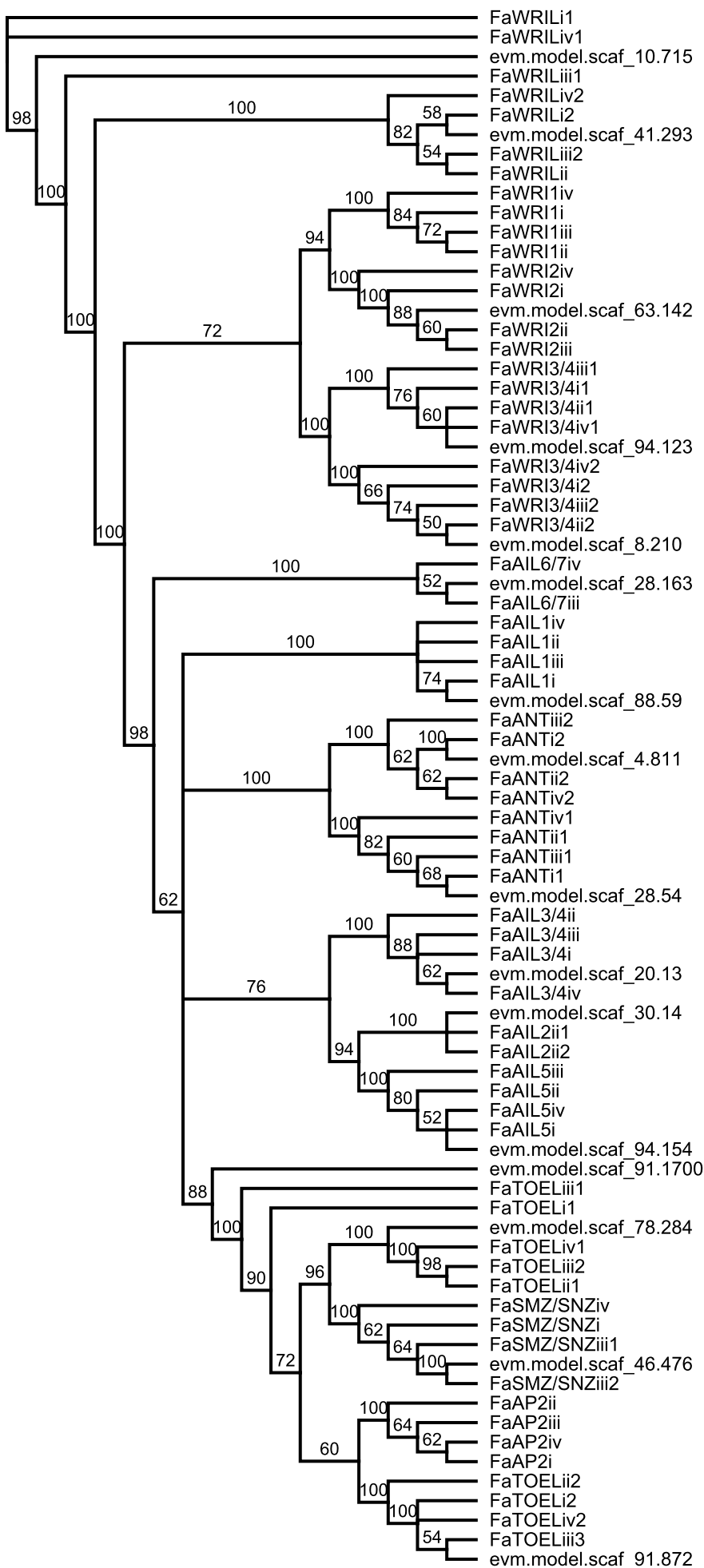

Supplement: Supplementary file 1 [file genes-12-00530-s001.zip › Figure s4.pdf]

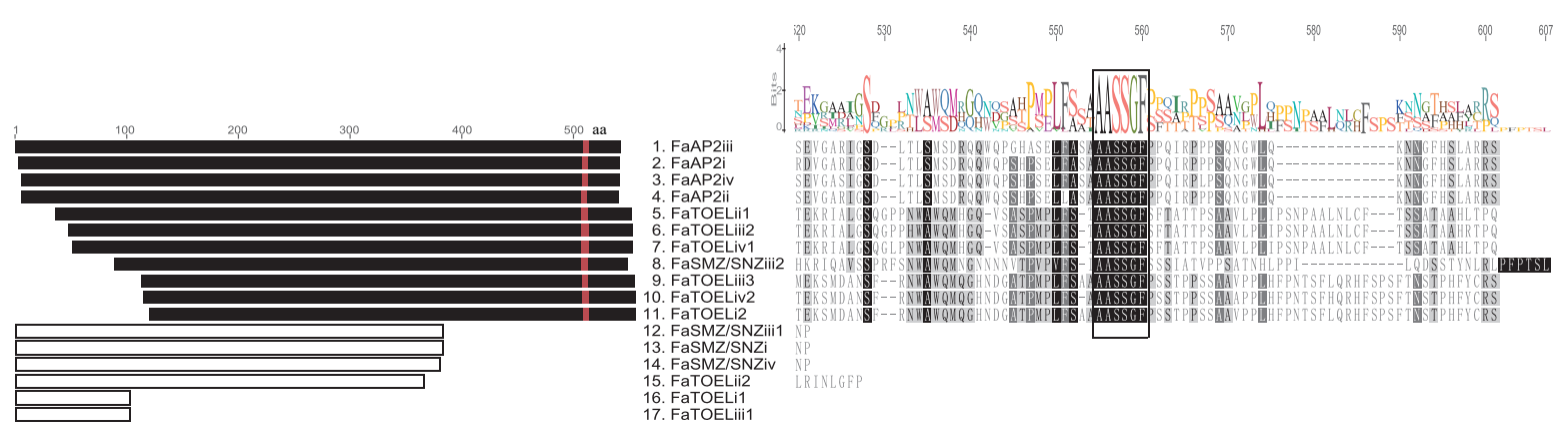

Supplement: Supplementary file 1 [file genes-12-00530-s001.zip › Figure S5.pdf]
